# Machine Learning for Mortality Prediction in Pediatric Myocarditis

**DOI:** 10.3389/fped.2021.644922

**Published:** 2021-04-23

**Authors:** Fu-Sheng Chou, Laxmi V. Ghimire

**Affiliations:** ^1^Department of Pediatrics, Loma Linda University School of Medicine, Loma Linda, CA, United States; ^2^Lakes Region General Hospital, Laconia, NH, United States

**Keywords:** pediatric myocarditis, mortality, machine learning, predictive modeling, random forest, extracorporeal membrane oxygenation

## Abstract

**Background:** Pediatric myocarditis is a rare disease. The etiologies are multiple. Mortality associated with the disease is 5–8%. Prognostic factors were identified with the use of national hospitalization databases. Applying these identified risk factors for mortality prediction has not been reported.

**Methods:** We used the Kids' Inpatient Database for this project. We manually curated fourteen variables as predictors of mortality based on the current knowledge of the disease, and compared performance of mortality prediction between linear regression models and a machine learning (ML) model. For ML, the random forest algorithm was chosen because of the categorical nature of the variables. Based on variable importance scores, a reduced model was also developed for comparison.

**Results:** We identified 4,144 patients from the database for randomization into the primary (for model development) and testing (for external validation) datasets. We found that the conventional logistic regression model had low sensitivity (~50%) despite high specificity (>95%) or overall accuracy. On the other hand, the ML model struck a good balance between sensitivity (89.9%) and specificity (85.8%). The reduced ML model with top five variables (mechanical ventilation, cardiac arrest, ECMO, acute kidney injury, ventricular fibrillation) were sufficient to approximate the prediction performance of the full model.

**Conclusions:** The ML algorithm performs superiorly when compared to the linear regression model for mortality prediction in pediatric myocarditis in this retrospective dataset. Prospective studies are warranted to further validate the applicability of our model in clinical settings.

## Introduction

Pediatric myocarditis is a rare disease of children. The etiologies are multiple, including viral, immune-mediated, and toxin-mediated (1). We, and others, have recently reported that in-hospital mortality associated with myocarditis is between 5 and 8% (2–4). The prevalence of pediatric myocarditis is bimodal, with higher prevalence in infancy and young childhood as well as in adolescence (2). The roles of sex and age as risk factors of mortality in pediatric myocarditis have been suggested. One study showed increased risk of mortality in younger children (3). Another study showed a higher prevalence of ventricular tachyarrhythmias, a predictor of mortality, in younger children (4, 5). Similarly, female sex has been associated with increased acuity of disease and mortaliy, although one study (Ghelani et al.) did not find a difference in the mortality or heart transplant rate between sex groups (2, 3, 5, 6).

In addition to sex and age, it has recently been reported that extracorporeal membrane oxygenation (ECMO) and tachyarrhythmias are associated with mortality (2–4, 7). Moreover, a recent report by Othman et al. studying 12,489 patients with pediatric myocarditis from 2002 to 2015 found ventricular arrhythmias were associated with significantly increased mortality (5). Using a national pediatric hospitalization database, we recently identified additional comorbidities that were associated with increased mortality and prolonged length of hospital stay; these comorbidities included brain injury (encephalopathy and/or anoxic or ischemic brain injury), acute kidney injury (AKI), sepsis and coagulopathy (4). These additional risk factors suggest that injury to distal vital organs are associated with increased severity of disease.

In this study, we aim to apply mortality-associated risk factors in mortality prediction for pediatric myocarditis. Given the interrelationship among cardiopulmonary compromise, aggressive cardiopulmonary support, and end-organ injury, which all have been identified as risk factors for mortality, the outcome predictions using a linear regression model may not be reliable. With the recent widespread adoption of machine learning (ML) algorithms in predictive model development, we aimed to compare the performance of traditional logistic regression models to that of ML-based models in pediatric myocarditis mortality prediction. We hypothesize that ML algorithm-based models outperform linear models.

## Materials and Methods

### Data Source and Record Selection

The Kids' Inpatient Database (KID) was used in this study. The KID is a survey-based public de-identified database of pediatric hospitalizations and is published every 3–4 years, with the latest release in 2016 (8). Datasets of 2003, 2006, 2009, 2012, and 2016 were purchased through the Healthcare Cost and Utilization Project (HCUP) online central distributor. Datasets from 2003 to 2012 contained International Classification of Diseases, Ninth Revision Clinical Modification (ICD-9-CM) diagnostic codes, whereas the 2016 dataset contained the ICD-10-CM diagnostic codes. Respective ICD-9-CM and ICD-10-CM codes for myocarditis and the comorbidity and procedure variables are listed in Supplementary Table 1. The nature of the KID as a publicly available de-identified database precludes the need for institutional board review.

**Figure 1:**
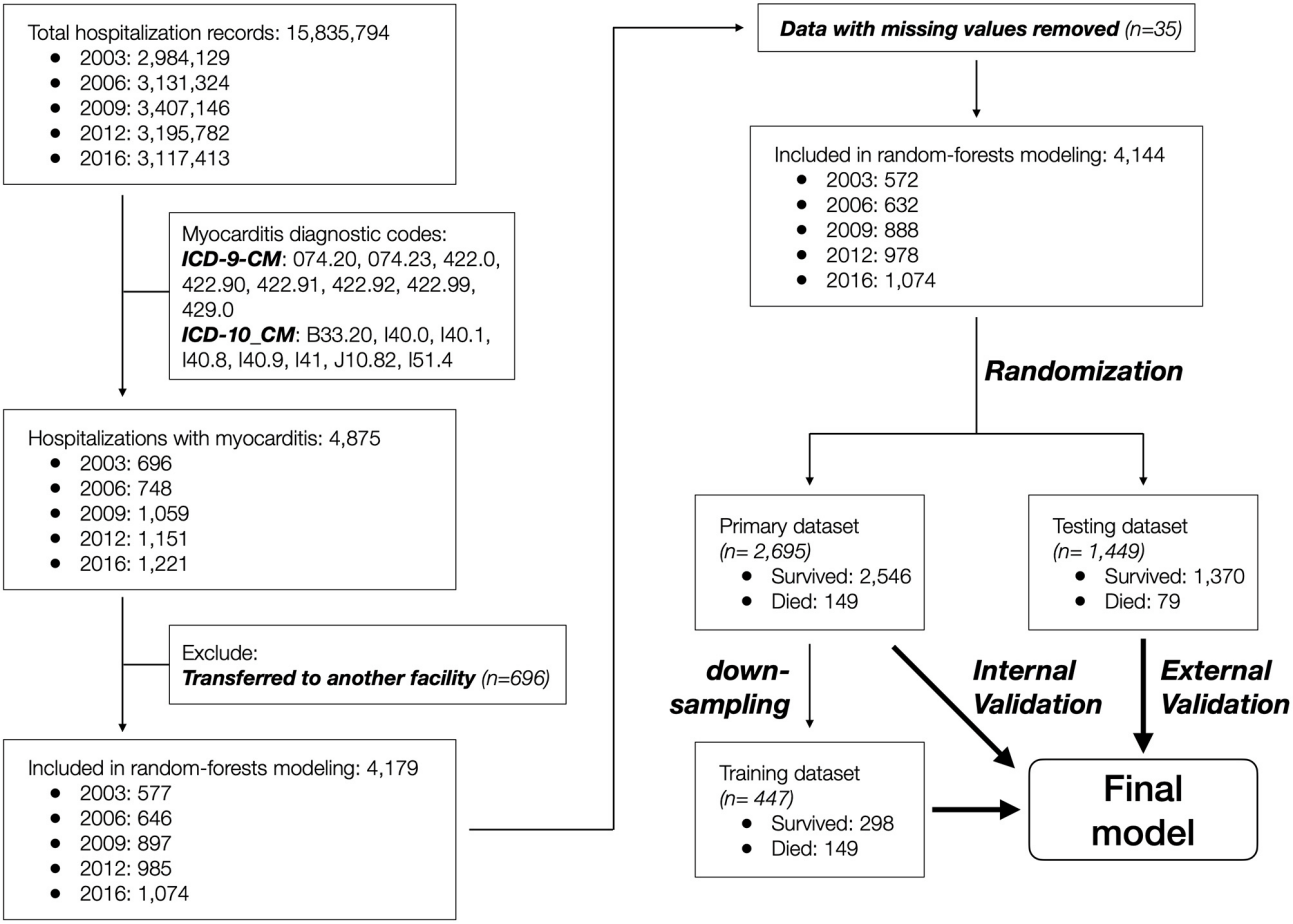
Modeling workflow with number of patients listed for each step.

Data records that contained a diagnostic code for myocarditis (*n* = 4,875) were included, and those that indicated inter-facility transfer (*n* = 696) or with missing sex and in-hospital mortality information (*n* = 35) were further excluded. The remaining 4,144 patient records were randomized into primary (*n* = 2,695, 65%) and testing (*n* = 1,449, 35%) datasets to achieve equal distribution of in-hospital mortality between the two groups. The primary dataset was used to develop the predictive model, and the testing dataset was used to externally validate the model. The patient selection and grouping process is summarized in Figure 1.

### Variable Selection

Initial comorbidity variable screening was performed as described (4). Briefly, datasets from 2003 to 2016 were combined, and the associated diagnostic and procedural ICD categorical codes were each tabulated for a random forest model training using all data records. The ICD codes were sorted by the variable importance scores. Comorbidity and procedural variables were selected by manual inspection based on current knowledge of myocarditis and the associated complications.

A table consisting of columns of selected comorbidity and procedural variables, as well as age, sex, and in-hospital mortality were constructed for survey-weighted logistic regression modeling and for ML model development. In-hospital mortality was considered as the outcome variable, whereas the remaining variables were considered as predictors.

### Descriptive Statistics

To determine whether each variable in question was associated with increased mortality, a survey-weighted χ^2^ test was performed. A *p*-value < 0.05 was considered as significant.

### Survey-Weighted Logistic Regression Modeling

Survey-weighted logistic regression modeling was performed with in-hospital mortality as the dependent variable and the selected comorbidity and the procedural variables as well as age and sex as the independent variables (Supplementary Table 2). Modeling was performed using the *survey* package for R (9). Coefficient estimates with 95% confidence intervals (CIs) were reported. The testing dataset was used for external validation, and probability was calculated using the inverse logit function.

### Supervised Machine Learning Model Development

Given the use of categorical variables as predictors for classification prediction, we chose the random forest algorithm for model development. Because the overall in-hospital mortality rate was 4.9% (4), random down-sampling was performed on the primary dataset to reach a 2:1 ratio between the survival (*n* = 298) and mortality (*n* = 149) groups, which comprised the training dataset. For model tuning, the *mtry* hyperparameter used to determine the number of variables available for splitting at each tree node was kept at a constant of 3, which was derived from taking the square root of total variable numbers (*n* = 14). *Minimum node size* of 1, 5, and 10, as well as *splitrule* using Gini index and extratrees, were supplied for comparison in model tuning. A 5-fold cross validation was performed to estimate model accuracy. The receiver operating curve (ROC) was used as model performance metrics when comparing models with various hyperparameter settings. After the final model was selected, internal and external validation were performed using the primary and the testing datasets, respectively. Modeling was performed using the *caret* and *ranger* packages in R 4.0.3 (10, 11). Codes are available upon request.

### Model Prediction

For both the logistical regression model and the ML model, a probability for in-hospital mortality of ≥50% was considered as positive. The area under the ROC was estimated. Model sensitivity, specificity, positive and negative predictive values, accuracy (the proportion of patients in whom predicted outcomes agreed with clinical outcomes), and Cohen's *kappa* (the proportion of accurate predictions not occurring by chance) were reported for comparison.

## Results

### Patient Record Description

A total of 4,875 patient records were identified to have a myocarditis diagnosis based on the ICD-9-CM (2003–2012 datasets) or ICD-10-CM (2016 dataset) codes. After removing 696 records with disposition indicating inter-facility transfer and 35 records with missing information regarding age and/or sex, the remaining 4,144 patient records were included in the study (Figure 1).

### Candidate Variable Selection

We recently reported risk factors for in-hospital mortality in pediatric myocarditis, including brain injury (encephalopathy, anoxic or ischemic injury), acute kidney injury, tachyarrhythmias, sepsis, coagulopathy, and ECMO need (4). These risk factors were included in the study.

**Figure 2:**
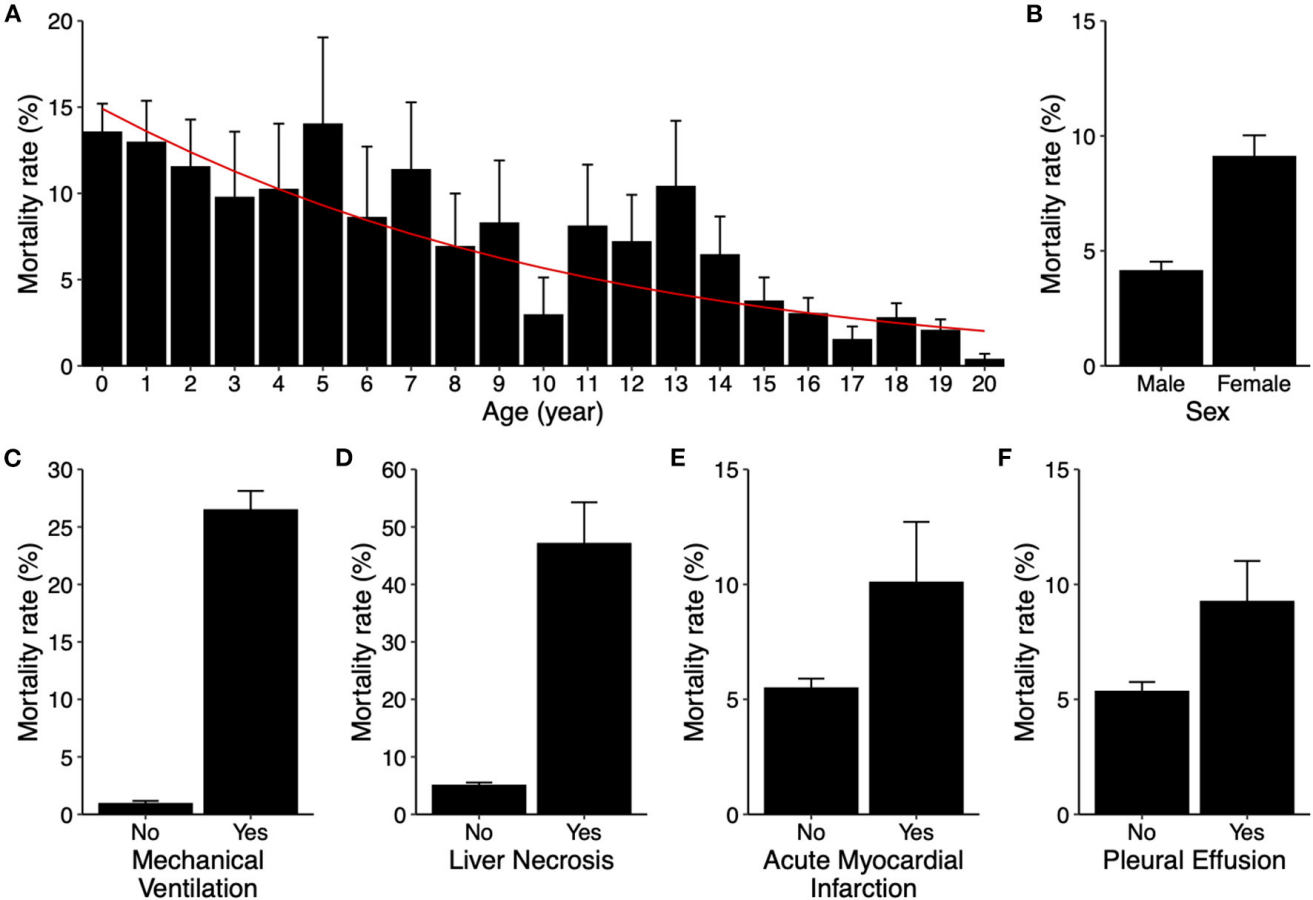
Risk factors for in-hospital mortality in pediatric myocarditis. **(A)** Mortality rates (bar graphs) stratified by age from 0 to 20 years old. Error bars indicate standard errors. *P*-value < 0.001. The red line indicates predicted mortality rate based on a univariable logistic regression model. **(B–F)** Mortality rates (bar graphs) stratified by sex (**B**, *p*-value < 0.001), mechanical ventilation use (**C**, *p*-value < 0.001), liver necrosis (**D**, *p*-value < 0.001), acute myocardial infarction (**E**, *p*-value = 0.296), and pleural effusion (**F**, *p*-value = 0.006). Error bars indicate standard errors.

We further assessed the roles of demographic factors, including age and sex, in mortality to determine inclusion of these two variables in model development. Survey-weighted analysis showed that mortality decreased as age increased, from 14% in infants to <0.5% in 20-year-old patients (*p*-value < 0.001). In a survey-weighted logistic regression model, the odds decreased by 10% for each increase in age by 1 year (Figure 2A). Female sex was associated with increased mortality (4.2 ± 0.4% in male vs. 9.1 ± 0.9% in female, *p*-value < 0.001, Figure 2B).

Additionally, variables that may implicate various degrees of cardiopulmonary compromise, including mechanical ventilation use, acute myocardial infarction (AMI), liver necrosis, and development of pleural effusion, were also assessed for inclusion. Except for AMI, the aforementioned variables were all associated with significant increases in mortality (Figures 2C–F). All of these risk factors were also included as variables in predictive model development.

In summary, a total of 14 variables were included, including age, sex, mechanical ventilation (MV), ECMO, cardiac arrest, ventricular fibrillation (VF), ventricular tachycardia (VT), AMI, pleural effusion, sepsis, coagulopathy, liver necrosis, AKI, and brain injury. The rates of occurrence for each variable in the primary and testing datasets with and without in-hospital mortality are listed in Table 1.

**Table 1:** Characteristics of the included patients for training and testing.

	**Primary Dataset**		**Testing Dataset**	
**In-hospital mortality**	**No**	**Yes**	**No**	**Yes**
Number	2,546	149	1,370	79
Age in year^*^	16 (8, 19)	5 (1, 15)	16 (8, 19)	3 (0, 11.5)
Female (%)	28.5%	54.4%	29.5%	36.7%
Mechanical ventilation	13.4%	86.6%	14.7%	83.5%
Cardiac arrest	1.4%	27.5%	1.7%	26.6%
Ventricular fibrillation	0.6%	10.7%	0.9%	8.9%
Ventricular tachycardia	6.1%	20.1%	7.7%	12.7%
Cardiomyopathy	15.9%	22.8%	15.8%	24.1%
Pleural effusion	6.5%	12.8%	7.7%	11.4%
Acute myocardial infarction	3.7%	5.4%	1.8%	6.3%
ECMO^†^	2.3%	29.5%	2.8%	29.1%
Sepsis	6.1%	24.2%	5.7%	29.1%
Coagulopathy^‡^	3.0%	26.2%	2.8%	27.8%
Liver necrosis	0.9%	7.4%	0.2%	16.5%
Acute kidney injury	5.9%	32.9%	6.3%	36.7%
Brain injury^§^	1.9%	15.4%	2.2%	19.0%

* *Median (25% quartile, 75% quartile)*.

† *ECMO, extracorporeal membrane oxygenation*.

‡ *Coagulopathy includes disseminated intravascular coagulation (defibrination syndrome), disseminated intravascular coagulation in newborn, acquired coagulation factor deficiency, and other unspecified coagulation defects*.

§ *Brain injury includes anoxic brain damage, unspecified encephalopathy, metabolic encephalopathy, other encephalopathy, intracranial hemorrhage (including extradural and subdural)*.

### Logistic Regression Modeling

**Figure 3:**
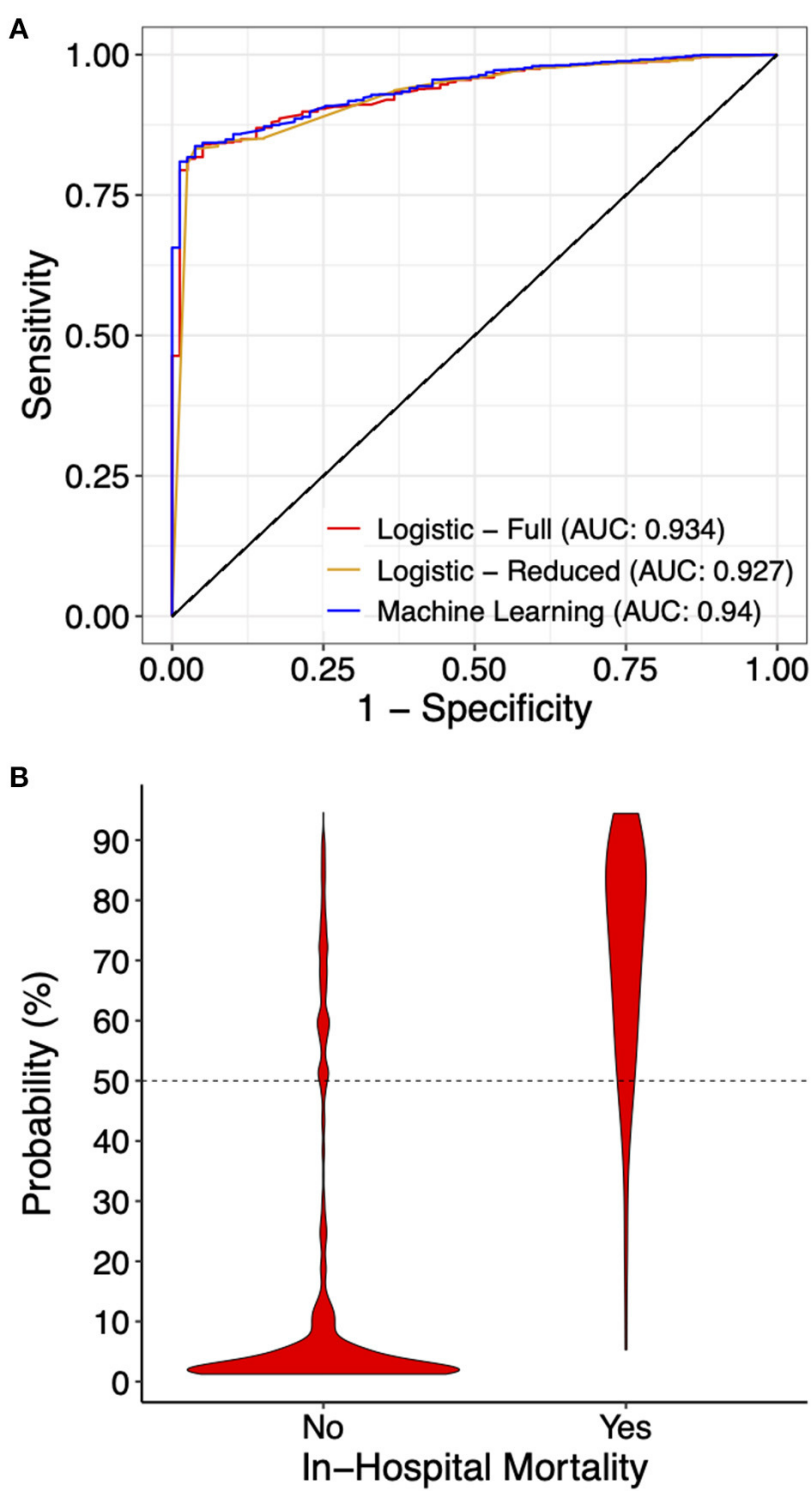
Prediction performance of the indicated models. **(A)** Receiver operating curves for the indicated models on the testing dataset. For the machine learning model, the full model with all 14 variables is presented. **(B)** A violin graph showing the distribution of the predicted probability with or without in-hospital mortality.

We first constructed a full multivariable logistic regression model (full model) including all 14 variables using the primary dataset, among which MV, cardiac arrest, VF, ECMO, coagulopathy, and AKI showed significant association with mortality after normalizing for all other variables (Supplementary Table 2). We then took the statistically significant variables (variables with coefficient *p*-value < 0.05) and constructed a reduced multivariable logistic regression model (reduced model). As expected, the reduced model had a lower Akaike information criteria score (695.1 in the reduced model, compared to 698.9 in the full model). Using both full and reduced models, we then calculated probability and assigned mortality prediction (probability ≥ 50%) for each data record in the primary and testing datasets, and performed model evaluation. The full model had an ROC area under curve (AUC) of 0.934, and the reduced model had an ROC AUC of 0.927 (Figure 3A). Both logistic models suffered from low sensitivity (around 50%) despite superior specificity (around 95%), and an overall accuracy close to 95% (Table 2).

**Table 2:** Model evaluation.

**Probability cutoff = 50%**	**Logistic - full model^†^**		**Logistic - reduced model^‡^**		**Machine learning - full model^†^**		**If predicted as all survived**
**Dataset**	**Primary**	**Testing**	**Primary**	**Testing**	**Primary**	**Testing**	**Testing**
Sensitivity	55.3%	52.5%	51.0%	50.0%	95.3%	89.9%	–
Specificity	95.9%	95.9%	95.3%	95.6%	87.7%	85.8%	94.5%
Positive Predictive Value	28.2%	26.6%	16.8%	20.3%	31.1%	26.7%	–
Negative Predictive Value	98.7%	98.6%	99.1%	98.8%	99.7%	99.3%	–
Accuracy	94.8%	94.7%	94.5%	94.6%	88.1%	86.0%	94.6%
Cohen's *kappa*	0.35	0.33	0.23	0.27	0.42	0.36	0

† *Including sex, age, mechanical ventilation, pleural effusion, cardiac arrest, extracorporeal membrane oxygenation, ventricular fibrillation, ventricular tachycardia, acute myocardial infarction, sepsis, coagulopathy, liver necrosis, acute kidney injury, and brain injury*.

‡ *Include mechanical ventilation, cardiac arrest, extracorporeal membrane oxygenation, ventricular fibrillation, coagulopathy, acute kidney injury*.

### Machine Learning Modeling

With a goal to develop a model for mortality prediction and the low sensitivity observed in the traditional logistic modeling approach, we turned our attention to ML. Given all variables being two-level categorical features, it was reasonable for us to choose the random forest algorithm, a decision tree-based algorithm, for model development.

Due to significant “class imbalance” as a result of a mortality rate of approximately 5% in pediatric myocarditis, we randomly down-sampled the survival group to a survival:mortality ratio of 2:1. The resultant 447 samples (298 survived and 149 died) were designated as the training dataset for use in model training and tuning (Figure 1). All 14 variables were used as predictors. In the final model, the distribution of mortality probability in the testing dataset showed a visually distinguishable separation between those with observed in-hospital mortality and those without (Figure 3B). Using a probability of ≥ 50% as cutoff for being predicted as having in-hospital mortality, the sensitivity was 95 and 90% with internal and external validation, respectively (Table 2). Using the testing data set, the specificity, positive predictive value, negative predictive value, and accuracy were 86, 27, 99, and 86%, respectively, all improved from the logistic models (Table 2). The ROC AUC was 0.940 (Figure 3A).

### Variable Importance

**Figure 4:**
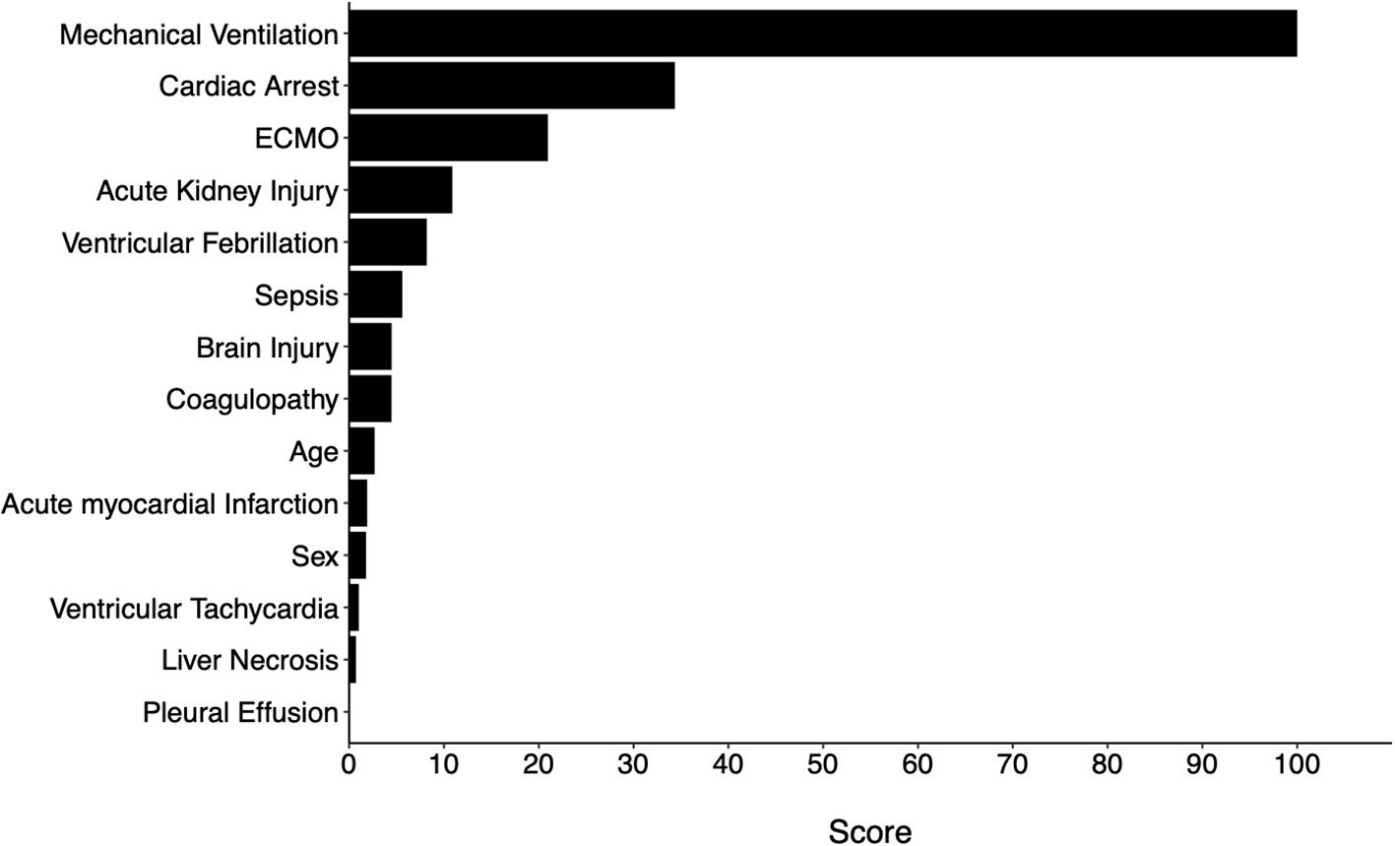
Variable importance scores from the full machine learning model.

Variable importance scores (VIS) were called from the final model. The top 5 variables with the highest scores were MV use, cardiac arrest, ECMO use, AKI, and VF (Figure 4). The predicted probability of mortality for each variable is listed in Table 3.

**Table 3:** Predicted probability of mortality with each variable (feature) alone using the machine learning model.

**Variable**	**Probability^†^**
None	5.0%
**Respiratory**	
Mechanical ventilation	51.1%
Pleural effusion	9.3%
**Cardiac**	
Cardiac arrest	75.1%
Ventricular fibrillation	37.4%
Ventricular tachycardia	6.8%
Acute myocardial infarction	6.2%
ECMO	40.8%
**Infection/inflammation**	
Sepsis	17.7%
Coagulopathy	11.7%
**Vital organ damage**	
Liver necrosis	7.1%
Acute kidney injury	34.4%
Brain injury	17.7%

† *Probability based on 0-year-old male patients*.

To further examine whether all 14 variables were needed for the same performance, two additional models were trained using the Top 5 and Top 10 variables based on VIS (Figure 4). The same hyperparameters for tuning and cross-validation were used. We found that the reduced models both had slightly inferior performance when compared to the full model, but there was no difference between the two reduced models (Table 4).

**Table 4:** Comparison of model performance on the testing dataset among various machine learning models trained using the indicated numbers of variables.

	**Testing Dataset**		
**Variables included**	**Top 5**	**Top 10**	**Top 14**
Sensitivity	88.6%	88.6%	89.9%
Specificity	84.8%	84.7%	85.8%
Positive Predictive Value	25.2%	25.0%	26.7%
Negative Predictive Value	99.2%	99.2%	99.3%
Accuracy	85.0%	84.9%	86.0%
*Kappa*	0.34	0.33	0.36
Number of patients that died but were predicted as survived	9	9	8
Number of patients that survived but were predicted as died	208	210	195

## Discussion

In this study, we aimed to develop a model for in-hospital mortality prediction among pediatric patients hospitalized for myocarditis. The traditional logistic regression models suffered from low sensitivity despite extremely high specificity and accuracy. In other words, logistic models were good at predicting patients who were likely to survive but performed poorly in predicting patients who were likely to die. On the contrary, the ML model had a good balance between sensitivity and specificity, and would be much more useful in clinical settings where predicting mortality is more crucial than predicting survival. A web app based on the full and reduced ML models has been developed for demonstration, and is accessible at https://neostat.shinyapps.io/myocarditis_ML_shiny/.

We chose to use the random forest algorithm because of the nature of the predictors, all of which are two-level categorial variables. For those datasets that include a mixture of two-level variables as well as variables with higher-order levels, random forest models may be biased toward having higher weights on multi-level variables. In those cases, interpretation of variable important scores may not be as reliable. The random forest algorithm also has a tendency of overfitting. Therefore, it is crucial to validate externally. Moreover, the classification output (yes or no for in-hospital mortality) and the corresponding AUC calculation may be arbitrary depending on the cutoff probability that is used to define class assignment.

Among top variables that predicted in-hospital mortality, mechanical ventilation and ECMO use were two procedures that indicated significant cardiopulmonary compromise. While VF and cardiac arrest usually indicated severe disease and moribund status of the patient, it was quite striking to us that AKI also carried considerable weight putting patients at a significant risk for mortality. We previously also showed that AKI was associated with increased length of hospital stay in pediatric patients with myocarditis by 2 folds (4). The significance of AKI in myocarditis prognosis has not been published in the pediatric population. However, a recent retrospective study assessing adult patients with myocarditis from 1996 to 2011 showed that AKI was associated with unfavorable outcomes. The caveat to our finding was the lack of information on the definition of AKI used in each institute. A better approach which was not feasible with the KID would be to use longitudinal measurements of urine output and kidney function tests in model development.

Based on variable importance scores, we selected top 5 and top 10 variables and developed additional models for comparison. We found that, although these additional models performed slightly inferiorly compared to the full model (with all 14 variables), the difference may not be clinically significant. Model performance was almost identical between the models developed with top 5 and top 10 variables. In other words, the probability of mortality may be satisfactorily approximated when any of these top 5 variables, namely MV, ECMO use, cardiac arrest, ventricular fibrillation, and AKI, are present. On the other hand, the full model may be used when other risk factors are used to estimate mortality risks.

We found that female patients were at increased risk for mortality, which was consistent with a recent report showing a higher percentage of female patients in the high-acuity group (6). Similarly, they also found high acuity at presentation in younger patients, consistent with our findings on the inverse association between age and mortality rate. However, these two demographic factors were not as important as other factors that were associated with significant cardiopulmonary compromise.

To our knowledge, there have been no prediction models published for mortality prediction in pediatric myocarditis. This report is the first to use identified risk factors for such purpose. In comparison, Wolf et al., in their single-center (a free standing children's hospital) cohort of 74 children with myocarditis, attempted to predict hemodynamic compromise using variables related to initial presentation (6). Hemodynamic compromise in their definition was a complex outcome including the need for inotropic or vasoactive medications, cardiopulmonary resuscitation, ECMO, ventricular assist devices (VAD), heart transplant, or those who died. They used two different linear models for prediction. The first model included the presence of tachycardia, tachypnea, creatinine, and cardiomegaly on chest radiograph as predictors, showing an AUC of 0.913. The second model added the presence of pericardial effusion to the above variables, with an AUC of 0.964. Although their prediction for hemodynamic compromise seems quite impressive, their model suffers from small sample size, single-center experience, extremely wide CIs for odds ratios suggesting variance inflation. Moreover, there was lack of external validation. Similarly, Othman et al. reported a multivariable linear regression model for mortality association, but there was no data on model performance as well as validation (5).

### Limitations

There are several limitations to this study. First, KID is an administrative database and the data were not specifically collected for research purposes. The diagnostic and procedural codes were entered for billing purposes. Therefore, incorrect or missing information may exist. For example, when inspecting the eight patients that were incorrectly predicted as survivors when the patients actually died, we found that ECMO was the only positive predictor for one patient (Supplementary Table 3). Based on clinical experience, this patient likely required MV, and have developed other comorbidities before ECMO support was initiated. The lack of MV and comorbidity codes is likely due to coding error. Additionally, some patients may receive a myocarditis code in the medical record as a result of clinical suspicion, rather than a true diagnosis of myocarditis with cardiac MRI or myocardial biopsy. Moreover, the use of the KID datasets precluded the ability to include cardiac medication, laboratory tests, and/or vital sign data in model development, which is one of the biggest weaknesses of this study.

### Conclusion

In this study, we developed a random forest model for mortality prediction in pediatric myocarditis with satisfactory performance. A ML-based model is superior to the traditional linear models. Future prospective studies are warranted to further validate this model for clinical use.

## Data Availability Statement

Publicly available datasets were analyzed in this study. This data can be found at: https://www.hcup-us.ahrq.gov/db/nation/kid/kiddbdocumentation.jsp.

## Ethics Statement

Ethical review and approval was not required for the study on human participants in accordance with the local legislation and institutional requirements. Written informed consent from the participants' legal guardian/next of kin was not required to participate in this study in accordance with the national legislation and the institutional requirements.

## Author Contributions

F-SC and LG conceptualized the study together and both contributed to interpretation of findings and manuscript writing. F-SC performed data organization and analysis. All authors contributed to the article and approved the submitted version.

## Conflict of Interest

The authors declare that the research was conducted in the absence of any commercial or financial relationships that could be construed as a potential conflict of interest.
