# Supplementary material for: Machine Learning for Mortality Prediction in Pediatric Myocarditis
Source: Front Pediatr. 2021 Apr 23;9:644922. doi: 10.3389/fped.2021.644922 (PMC8102689; doi:10.3389/fped.2021.644922)
Supplement: Supplementary file 1 [file Table_1.pdf]

Supplemental Table 1. International classifications of diseases codes used to assemble each variable

Variable	ICD-9 Codes	ICD-10 Codes
**Myocarditis**	074.20, 074.23, 422.0, 422.99, 422.90, 422.91, 422.92, 429.0	B33.20, J10.82, I40.0, I40.1, I40.8, I40.9, I41, I51.4
**Mechanical ventilation***	960.4, 967.xx	0BH.18EZ, 5A1.935Z, 5A1.945Z, 5A1.955Z
**Cardiac arrest**	427.5	I46.9
**Ventricular fibrillation**	427.41	I49.01
**Ventricular tachycardia**	427.1	I47.2
**Cardiomyopathy**	425.3x, 425.4x, 425.8x, 425.9x	I42.4x, I42.5x, I42.7x, I42.8x, I43
**Pleural effusion**	511.1x, 511.8x, 511.9x	J90
**Acute myocardial infarction**	410.xx	I21.xx
**ECMO†**	396.5	5A1.5223
**Sepsis**	995.91, 995.92, 771.81	A41.9, R65.2, P36.9
**Coagulopathy‡**	286.6, 286.7, 286.9, 776.2	D65, D68.4, D68.8, D68.9, P60
**Liver necrosis**	570	K76.2
**Acute kidney injury**	584.xx	N17.xx
**Brain injury§**	348.1, 348.30, 348.31, 348.39, 432.xx, 852.20, 852.22	G93.1, G93.40, G93.49, I62.00, I62.1, I62.9, S06.5

*Procedural codes
†ECMO: extracorporeal membrane oxygenation.
‡Coagulopathy includes disseminated intravascular coagulation (defibrination syndrome), disseminated intravascular coagulation in newborn, acquired coagulation factor deficiency, and other unspecified coagulation defects.
§Brain injury includes anoxic brain damage, unspecified encephalopathy, metabolic encephalopathy, other encephalopathy, intracranial hemorrhage (including extradural and subdural)

Supplemental Table 2. Odds ratio of each variable in a multiple logistic model for mortality in pediatric myocarditis

**Risk factors**	**Odds ratio**	**95% Confidence Interval**	**P Value**
Demographics			
Age	1.0	0.9 - 1.0	0.138
Sex	1.4	0.9 - 2.2	0.116
Respiratory			
Mechanical ventilation	15.8	8.5 - 29.5	< 0.001
Pleural effusion	0.6	0.3 - 1.18	0.129
Cardiac			
Cardiac arrest	6.2	3.1 - 12.5	< 0.001
Ventricular fibrillation	5.1	1.6 - 16.4	0.007
Ventricular tachycardia	1.7	0.9 - 3.1	0.078
Acute myocardial infarction	1.3	0.5 - 3.6	0.567
ECMO	2.4	1.3 - 4.5	0.007
Infection/Inflammation			
Sepsis	1.5	0.8 - 2.8	0.217
Coagulopathy	2.5	1.5 - 4.3	0.007
Vital Organ Damage			
Liver necrosis	1.2	0.5 - 3.2	0.645
Acute kidney injury	2.2	1.3 - 3.8	0.004
Brain injury	1.4	0.7 - 3.0	0.365

Supplemental Table 3. Characteristics of mis-classified patients that died but predicted as survived from the testing dataset

Patient	#1	#2	#3	#4	#5	#6	#7	#8
Demographics								
Age	0	0	1	1	8	9	12	14
Female				+				
Respiratory								
Mechanical ventilation	+	+						
Pleural effusion							+	
Cardiac								
Cardiac arrest								
Ventricular fibrillation								
Ventricular tachycardia				+			+	
Acute myocardial infarction		+						
ECMO				+		+		
Infection/Inflammation								
Sepsis			+					
Coagulopathy								+

End-Organ Damage								
Liver necrosis								+
Acute kidney injury					+			+
Brain injury								
**Predicted mortality probability (%)**	**40.0**	**44.7**	**18.7**	**47.7**	**24.0**	**44.0**	**5.7**	**34.9**
